# Effects of Daily Intake of Rosehip Extract on Low-Density Lipoprotein Cholesterol and Blood Glucose Levels: A Systematic Review

**DOI:** 10.7759/cureus.51225

**Published:** 2023-12-28

**Authors:** Malachy Belkhelladi

**Affiliations:** 1 Biochemistry, McGill University, Montreal, CAN

**Keywords:** cardiovascular health, insulin sensitivity, low-density lipoprotein cholesterol, natural treatment, metabolic health, dietary supplements, rosa canina

## Abstract

This systematic review evaluates the effects of the daily intake of rosehip extract on low-density lipoprotein cholesterol (LDL-C) and blood glucose levels. It synthesizes findings from randomized clinical trials focusing on cardiovascular and metabolic health outcomes. The review includes studies employing various forms of rosehip supplementation, assessing primary outcomes such as LDL-C, HDL-C, total cholesterol, triglycerides, fasting blood glucose (FBG), and glycated hemoglobin (HbA1c). Secondary outcomes, such as body weight, BMI, and blood pressure, are also considered. The paper discusses the potential mechanisms of rosehip’s action, including modulation of peroxisome proliferator-activated receptors and effects on various metabolic pathways. The results indicate mixed effects on lipid profiles and blood glucose levels, with some studies showing significant benefits. This review underscores the need for further research to confirm optimal dosages, treatment durations, and rosehip's efficacy in diverse populations, considering its favorable safety profile. The findings suggest the potential of rosehip extract as a complementary agent in managing cardiometabolic risk factors.

## Introduction and background

Effective management of low-density lipoprotein cholesterol (LDL-C) and blood glucose levels is crucial to cardiovascular and metabolic health. LDL-C is primarily responsible for transporting cholesterol to various tissues throughout the body. However, high levels of LDL-C can lead to the accumulation of cholesterol in arterial walls [1], significantly contributing to the development of atherosclerosis, a central pathological process in cardiovascular disease (CVD) [2], the leading cause of death globally [3]. Concurrently, uncontrolled blood glucose levels significantly contribute to the development and progression of metabolic diseases, including type 2 diabetes mellitus (T2DM), which has catapulted to the ninth leading cause of death globally, a significant escalation from its eighteenth position in 1990 [3]. Elevated blood glucose levels are also a significant independent risk factor for cardiovascular mortality [4]. The global prevalence of metabolic diseases such as T2DM, hypertension, and non-alcoholic fatty liver disease (NAFLD) has increased notably over the past two decades, with metabolic syndrome prevalence reaching almost 20% in adults over the age of 18 [5], demanding urgent attention.

The economic burden of these diseases is staggering. In the United States alone, cardiovascular diseases accounted for 928,741 deaths in 2020, with associated costs reaching $407.3 billion between 2018 and 2019 [6]. Diabetes, a direct consequence of unmanaged blood glucose levels, incurred an estimated cost of $327 billion in 2017 in the U.S. [7]. Furthermore, the global economic impact of obesity is projected to hit $4.32 trillion annually by 2035, highlighting the financial weight of metabolic diseases [8].

Beyond cholesterol and glucose levels, other factors play a significant role in influencing cardiovascular and metabolic health. Body weight and abdominal fat, for instance, are closely linked with both LDL-C and blood glucose levels [9,10]. Obesity, particularly central obesity, is a known risk factor for the development of insulin resistance, dyslipidemia, and hypertension [11]. These interrelated factors further contribute to the overall risk of cardiovascular and metabolic diseases. Therefore, a holistic approach that addresses all these variables is essential in the management and prevention of these globally prevalent health issues.

Given this context, there has been a growing interest in natural, plant-based therapies that have historically been used for various ailments. These compounds have demonstrated potential efficacy in treating a wide range of medical conditions, including Alzheimer's [12], arthritis [13], cardiovascular diseases [14], and certain cancers [15]. One such promising natural compound is rosehip extract (RH). Produced from the pressed fruit of the rosehip (Rosa Canina L.) plant, RH is rich in essential fatty acids such as linolenic acid, linoleic acid, and oleic acid. It finds applications in diverse sectors like the pharmaceutical, cosmetic, and food industries [16]. While preliminary research has explored the potential metabolic effects of RH, a comprehensive synthesis and evaluation of these studies, particularly regarding their impact on metabolic markers such as LDL and blood glucose levels, remains absent. Given the escalating prevalence of CVDs and metabolic disorders, the consequential socio-economic burden, and the limitations of current preventive treatments, this review aims to provide a comprehensive assessment of RH as a potential therapeutic intervention for the control of LDL-C and blood glucose levels.

## Review

Methods

Search Strategy

**Figure 1:**
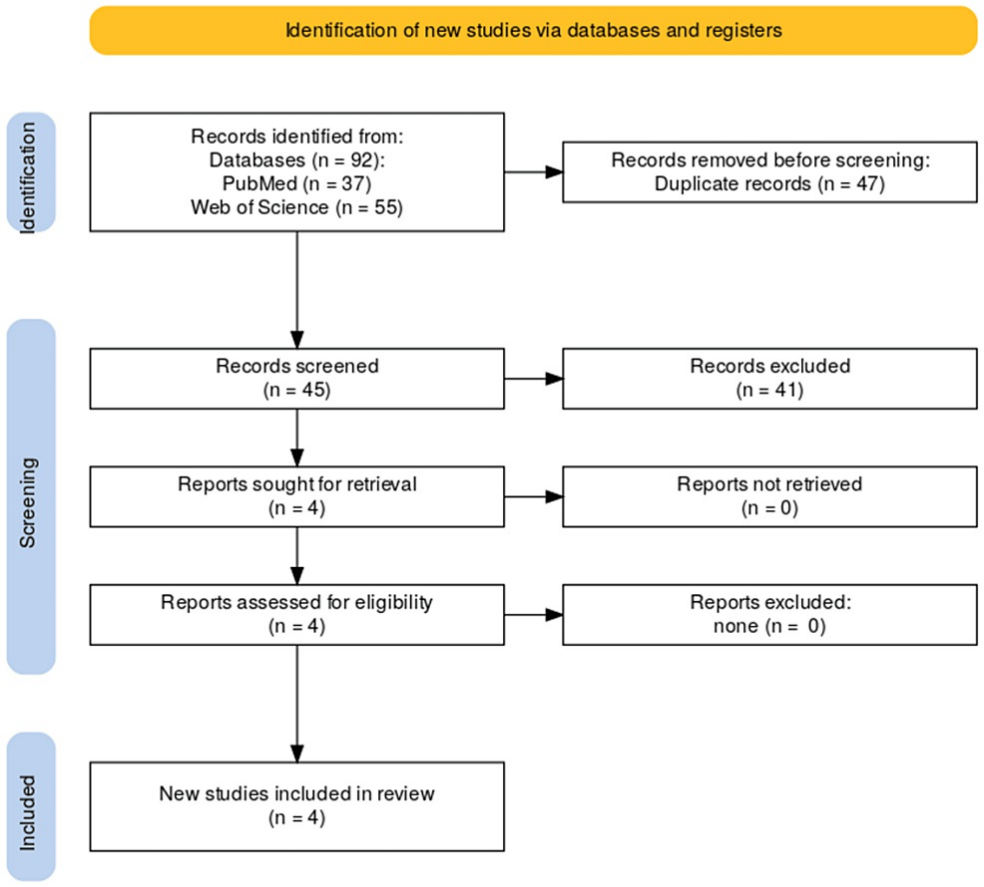
Preferred reporting items for systematic reviews and meta-analyses flow diagram of search results.

The web of Science and PubMed databases were searched for articles published in print or electronically before November 2023. Additional records were identified through Google Scholar. The search strategy included the terms: rosehip, rosa canina, rosehip oil, rosehip extract, diabetes, obese, cardiovascular, LDL, cholesterol, and glucose. The preferred reporting items for systematic reviews and meta-analyses flow diagram of the search results is shown in Figure 1 [17].

Selection Criteria

The inclusion criteria for the selected articles were: (1) randomized double-blind clinical trials; (2) studies available in English; (3) participants over the age of 18; (4) oral administration of RH; and (5) studies measuring LDL cholesterol; and (6) studies assessing blood glucose levels.

The exclusion criteria for the selected articles were: (1) studies not published in English; (2) studies that do not provide original data, such as reviews, editorials, or opinion pieces; and (3) studies not measuring LDL or blood glucose levels. (4) studies with a high risk of bias and (5) studies where full text was not available were also excluded.

Data Analysis and Extraction

Once articles meeting the inclusion criteria were identified, the lead author independently extracted information from each article. First, the titles and abstracts of each article were screened for relevance. Then, full articles were reviewed, and data was extracted. The main data points extracted from each article were: study design, patient characteristics, and primary and secondary findings. Reference lists of all included papers were reviewed, with no new manuscripts identified. The screening tool, Rayyan.ai, was employed to organize and screen studies.

Risk of Bias Assessment

In accordance with the Cochrane Handbook for systematic reviews of interventions, the risk of bias (RoB) 2 tool was used to assess methodological quality [18]. The analysis was conducted by the lead author, who independently assessed the risk of bias for each included study. A modified version of the tool was used for the assessment of randomized crossover trials.

The RoB 2 tool assessed the randomization of the assignment for the following domains: selection bias (random sequence generation, allocation concealment); performance bias (blinding of participants and personnel); detection bias (blinding of outcome assessment); attrition bias (incomplete outcome data); reporting bias (selective outcome reporting). A judgment was made depending on the following criteria: low risk of bias, unclear risk of bias, and high risk of bias. Low risk of bias meant when all domains were at low risk. Unclear risk of bias meant when one or more domains were at unclear risk, but none were at a high risk of bias. High risk of bias meant when one or more domains were at high risk of bias or multiple domains were at unclear risk that these reduced confidence in the results.

Results

Initial database searches identified 45 unique articles; four articles were selected for detailed review (Figure 1). Four articles were included in the final review (Table 1).

**Table 1:** Study characteristics

Article	Study Design	Study Duration	Sample Size	Mean Age	Treatment Dose	Risk of Bias	Effect on LDL	Effect on Blood Glucose	Secondary Findings
Andersson et al., 2011 [19]	Randomized double-blind crossover trial	6 weeks	31 (9M/22F)	57.0	500ml drink containing 40g of rosehip powder	Low	RH group experienced a significant decrease in LDL-C and LDL/HDL ratio.	No significant effects were observed on HbA1c or FBG.	RH group had a significant reduction in systolic blood pressure and Reynold's cardiovascular risk score compared to the control group.
Nagatomo et al., 2015 [20]	RCT	12 weeks	32 (16M/16F)	50.4	100mg tablet of rosehip extract daily	Low	No significant difference in lipid outcomes was observed between groups.	The RH had no significant effect on levels of FBG compared to control.	The RH group experienced a significant decrease in body weight, BMI, abdominal visceral fat, and abdominal total fat area compared to the control group.
Dabaghian et al., 2015 [21]	RCT	12 weeks	48 (21M/27F)	56.1	750mg tablet of rosehip extract 2x daily	Some concerns	RH group experienced a significant decrease in the total cholesterol/HDL-C ratio but no significant effect on LDL-C compared to the control.	The RH group demonstrated a significant reduction in fasting plasma glucose compared to placebo.	No significant secondary outcomes were reported.
Mehrzadi et al., 2020 [22]	RCT	12 weeks	150 (35M/115F)	54.0	225mg tablet of rosehip extract 2x daily	Low	No significant difference in lipid outcomes was observed between groups.	The RH group demonstrated a significant reduction in fasting plasma glucose and HbA1c compared to placebo and was as effective as metformin.	No significant secondary outcomes were reported.

Characteristics of Included Studies

The first clinical trial by Andersson et al. [19] evaluated the effects of rosehip extract on risk markers of type 2 diabetes and cardiovascular disease. The study had a total sample size of 31 obese individuals, defined as having a body mass index (BMI) > 30. Participants consisted of nine males and 22 females, with a mean age of 57.0 years. Nine participants were on anti-hypertensive medications, and eight were on lipid-lowering statins. The clinical trial was a randomized, double-blind crossover trial lasting 14 weeks. Patients were assigned to one of two groups. One group received the RH first, then the placebo second; the other group proceeded in the reverse order. Each treatment period lasted six weeks, with a two-week "washout" period in between. Participants in the treatment group drank 500 ml of a drink containing 40g of ground rosehip powder daily; the placebo was an identical drink with the exception of the rosehip powder. The primary outcomes measured were body weight, glucose tolerance, blood pressure, and blood lipids. Markers of inflammation, such as C-reactive protein and adiponectin, were also measured. Additionally, researchers calculated Reynold’s cardiovascular risk score, a measure of the 10-year risk of cardiovascular events, for all participants pre- and post-intervention.

The second trial by Nagatomo et al. [20] focused on the effect of RH on body fat, specifically abdominal body fat. Enrolling a sample size of 32 pre-obese participants, defined as having a BMI between 25 and 30. The gender distribution consisted of 16 males and 16 females, with a mean age of 50.4 years of age. Participants in the RCT took a 100mg tablet of rosehip extract once daily for 12 weeks, with measurements taken at 0, 4, 8, and 12 weeks. Total, abdominal, subcutaneous, and visceral fat measurements were taken by computed tomography (CT) scans using fat measurement software. The primary outcomes measured were body weight, body fat percentage, and abdominal visceral, subcutaneous, and total fat areas. Lipid levels, blood glucose levels, blood pressure, and heart rate were also measured.

The third clinical trial by Dabaghian et al. [21] assessed the metabolic effects of RH in patients with T2DM and fasting blood glucose levels of 130-250 mg/dl. The RCT employed a sample of 48 patients, including 21 males and 27 females between the ages of 35 and 60, with a mean age of 56.1 years. Participants received either 750 mg capsules of R. canina fruit extract or placebo twice daily for three months. The primary outcomes measured were fasting blood glucose (FBG), and postprandial blood glucose (PBG), and glycosylated hemoglobin (HbA1c). Secondary outcomes included lipid profile hepatic and renal function.

In the most recent clinical trial by Mehrzadi et al. [22], researchers examined the effect of RH on markers of glucose control. The cohort consisted of 150 participants diagnosed with type 2 diabetes, including 35 males and 115 females, with a mean age of 54.0 years. The randomized controlled trial lasted 12 weeks and consisted of three groups of patients on the anti-hyperglycemic medication 10mg Glyburide in combination with twice-daily herbal combination tablets, which included 225 mg rosehip extract, 1000 mg metformin, or a placebo. The primary outcomes measured were fasting plasma glucose and glycated hemoglobin (HbA1c). Blood lipid levels were also measured.

Efficacy

Lipid levels:* *All four of the analyzed studies assessed the effect of RH on lipid levels via serum analyses of LDL-C, HDL-C, total cholesterol (total-cho), triglycerides (TG), and various lipid ratios such as LDL-C/HDL-C and total cholesterol/HDL-C.

Andersson et al. [19] reported a significant reduction in total cholesterol (-4.9%) and LDL cholesterol (-6.0%) from baseline, while the LDL-C/HDL-C ratio decreased by 6.5%, with rosehip intake demonstrating a significant decrease compared to controls in each of these measures. Notably, excluding participants on statin treatment accentuated these effects, with researchers observing a 5.5% reduction in total cholesterol and an 8.6% decrease in LDL cholesterol.

However, this positive trend was not universally observed. Studies by Nagatomo et al. [20], Dabaghian et al. [21], and Mehrzadi et al. [22] reported no significant effects on triglycerides, HDL, LDL, or total cholesterol levels. Intriguingly, Dabaghian et al. noted a significant reduction in the total cholesterol/HDL-C ratio (-17.4%) compared to baseline and significantly decreased compared to controls, underscoring a potential large specific impact of rosehip on this ratio, despite no changes in the individual lipid parameters.

Blood glucose levels: The primary methods used to assess rosehip’s effect on blood glucose were fasting blood glucose and glycated hemoglobin. FBG measures blood glucose after an overnight fast, reflecting short-term glucose control. HbA1c provides an average of blood glucose levels over approximately three months, offering a longer-term perspective.

In assessing the effects of rosehip extract on blood glucose levels, the studies show mixed results. Andersson et al. [19] and Nagatomo et al. [20] reported no significant effects on fasting plasma glucose (FBG). HbA1c was not measured in Nagatomo et al.'s study. Dabaghian et al. [21] observed a significant decrease in FBG (-16.2%) in the rosehip group compared to baseline, with a significantly greater reduction compared to the placebo group. No significant effect on HbA1c levels was reported.

Mehrzadi et al. [22] also reported a notable decrease in FBG (-20.4%) in the RH group, with the herbal treatment being as effective as metformin. They also found a significant reduction in HbA1c (-13.5%), again mirroring metformin's effectiveness. These results were significantly greater than those in the placebo group.

Secondary findings:* *The secondary findings in the reviewed studies present varied outcomes. Andersson et al. [19] observed no significant effects on body weight or BMI but reported a significant reduction in systolic blood pressure (-3.4%) and a 17% decrease in the Reynolds risk score for cardiovascular disease (CVD), both greater than in the placebo group. This study was unique in calculating the Reynolds risk score.

Nagatomo et al. [20] found significant decreases in abdominal total fat area (-6.4%), abdominal visceral fat area (-10.4%), body weight (-2.0%), and BMI (-2.0%) after rosehip intake, with reductions greater than those in the placebo group. This was the only study assessing changes in body fat. No significant effect on systolic blood pressure was noted. Dabaghian et al. [21] and Mehrzadi et al. [22] did not assess any secondary measurements.

Safety and tolerability:* *In terms of safety and tolerability, Andersson et al. [19] reported that both rose hip and control drinks were well-tolerated. However, mild gastrointestinal issues were more common in the rose hip group, including loose stools and flatulence. Nagatomo et al. [20] found no adverse effects from rosehip tablet intake, with no significant abnormalities in physical, biochemical, hematological, or urinalysis parameters. Dabaghian et al. [21] also noted the good tolerability of R. canina, with mild, self-limiting gastrointestinal complaints being the most common adverse effects. Mehrzadi et al. [22] reported no adverse impacts on renal and hepatic function with the short-term use of rosehip formulation. This indicates a generally favorable safety profile for rosehip extracts, with mild gastrointestinal issues being the most noted side effect.

Discussion

The current systematic review aimed to provide a comprehensive assessment of rosehip (RH) as a potential therapeutic intervention for the control of low-density lipoprotein cholesterol (LDL-C) and blood glucose levels.

In assessing the efficacy of rosehip extract, noteworthy positive trends were observed. For instance, Andersson et al. demonstrated significant reductions in total cholesterol and LDL cholesterol, with the exclusion of statin users intensifying these effects [19]. Dabaghian et al. also reported a notable decrease in the total cholesterol/HDL-C ratio [21]. In terms of blood glucose regulation, both Dabaghian et al. and Mehrzadi et al. observed significant reductions in fasting blood glucose and HbA1c, showing parallels in efficacy to metformin [21,22]. In addition to these primary findings, RH demonstrated some impact on secondary outcomes. Andersson et al. noted a reduction in systolic blood pressure and the Reynolds risk score for cardiovascular disease. Conversely, Nagatomo et al. reported decreases in abdominal fat, body weight, and BMI [20]. These secondary findings further underscore the potential broader health benefits of rosehip extract.

Despite these encouraging findings, not all studies echoed these results. Nagatomo et al., for example, found no significant changes in lipid or blood glucose levels, and a consistent effect on LDL-C was specifically noted only in Andersson et al.’s trial. The discrepancies among these findings can be attributed to the diversity of study designs. Andersson et al. utilized a rosehip drink, while the other studies employed different doses of tablets, hinting at a potential influence of the form of RH supplementation on its efficacy. Additionally, the study populations varied: Andersson et al. and Nagatomo et al. included obese and pre-obese participants, whereas Mehrzadi et al. and Dabaghian et al. focused solely on patients with type II diabetes. These demographic differences are crucial, as they could influence how RH affects blood glucose markers and lipid profiles. The variation in study duration, sample size, geographic location of the studies, and preparation of the extract could also have played a role in the differing outcomes. Understanding these nuances is essential for interpreting the data and guiding future research directions in this area.

The mechanisms behind these findings have been explored in a plethora of rodent studies. The lipid-lowering actions of RH may be attributed to the modulation of peroxisome proliferator-activated receptors (PPARs), with studies indicating RH may increase energy expenditure and decrease energy absorption by augmenting PPARα expression [23], promoting fatty acid oxidation, inhibiting PPARγ expression [24], and reducing lipid storage in adipose tissues. Additionally, the flavonoids and antioxidants present in RH are known to inhibit apolipoprotein B-100 synthesis, thereby reducing LDL production [25]. These changes in PPAR expression and effects on ApoB influence lipids, which may explain the observed effects on lipid profiles in Andersson et al. and Dabaghian et al. [21]. Furthermore, the rodent models suggest a dose-dependent effect, with a dose of 250mg/kg producing the most optimal outcomes [26]. The discrepancy in dosages from rodent to human trials may clarify the limited effects observed on lipid profiles in humans.

Regarding blood glucose regulation, RH has been shown to enhance insulin sensitivity [27,28] and inhibit gluconeogenesis while also regenerating pancreatic beta cells [29]. This elevated β-cell may lead to enhanced insulin sensitivity [30], which could facilitate reduced blood glucose levels. These effects collectively contribute to improved glucose homeostasis. The relationship between glycemic control and complications of type 2 diabetes mellitus (T2DM) is well established. The landmark UK Prospective Diabetes Study (UKPDS) [31] demonstrated that every 1% reduction in HbA1c is associated with significant reductions in the risk of diabetic complications. Maintaining good glycemic control, typically measured by levels of glycated hemoglobin (HbA1c), is crucial to delaying or preventing the development of diabetes-related complications [32].

RH's influence on body weight and composition seems to be a consequence of heightened whole-body energy expenditure, notably through the browning of white adipose tissue driven by genes like UCP1, as demonstrated in rodent models [28]. The mechanisms underlying the observed reduction in systolic blood pressure in the study by Andersson et al. remain less defined. This reduction might be an indirect result of the decrease in lipid levels, aligning with the established relationship between lipid management and blood pressure control [33]. Further investigation is needed to elucidate these complex interactions and the broader implications for metabolic health.

The results from these clinical trials suggest that rosehip extract has the potential to be a complementary therapeutic agent in managing cardiometabolic risk factors, particularly for patients at risk of developing T2DM or CVDs. Its influence on lipid metabolism, body weight reduction, and glucose regulation makes it a promising candidate. However, it is important to re-iterate that the results across studies were mixed, indicating a need for more extensive trials to ascertain optimal dosages, treatment durations, and its efficacy in diverse populations. Given its favorable safety profile, clinicians may consider these findings while carefully weighing them against individual patient needs and circumstances in the context of alternative or supplementary treatment options. Clarifying trials are essential to fully understanding the scope of RH's therapeutic potential.

Some limitations to this review must be noted. Potential biases may have been introduced during the review process, particularly due to the specific inclusion and exclusion criteria set forth. The inclusion criteria allowed for a broad scope of study designs, thereby increasing the volume of data available for analysis. However, this broad scope also introduced a high degree of heterogeneity among the studies, encompassing variables such as study design, sample size, participant demographics, and measured variables. This diversity might limit the ability to draw clear comparisons and may affect the generalizability of our review's results. It is crucial for future research to address these limitations, ensuring a more standardized approach and enhanced clarity on RH’s role as a preventative treatment for CVDs and T2DM.

## Conclusions

In conclusion, some evidence suggests that oral supplementation with rosehip extract may lead to a decrease in LDL-C and fasting blood glucose, with additional effects on cardiovascular risk markers such as systolic blood pressure, visceral body fat, and BMI. Yet, as compelling as these findings might be, it’s essential to approach them with caution. The heterogeneity of the studies and conflicting results inject a degree of uncertainty into the findings. While the existing data provide some promising insights into the potential use of RH as a preventative treatment for CVDs and T2DM, more rigorously designed studies are needed further to explore the therapeutic use of RO in oral preparations.

Furthermore, it’s critical to recognize that while RH may provide a host of metabolic and cardiovascular benefits, it does not operate in isolation. It forms part of a broader ecosystem of factors contributing to the development and treatment of diabetes. These factors include the individual’s age, health status, medications, and lifestyle factors, such as smoking and diet. These elements need to be considered in future research on RH’s potential therapeutic uses.
